# Salvage radiotherapy for locally recurrent cervical and endometrial carcinoma: clinical outcomes and toxicities

**DOI:** 10.1186/s12885-024-12617-8

**Published:** 2024-07-19

**Authors:** Hui Cong, Xiaojing Yang, Zhaobin Li, Zhen Li, Shuchen Lin, Wei Jiang, Jie Fu

**Affiliations:** 1 Department of Radiation Oncology, Shanghai Sixth People’s Hospital Affiliated to Shanghai Jiao Tong University School of Medicine, No. 600, Yishan Road, Shanghai, 200233 China; 2 Department of Gynecology, The Obstetrics and Gynecology Hospital of Fudan University, 419 Fangxie Road, Shanghai, 200011 China

**Keywords:** Gynecological cancer, Vaginal recurrence, Salvage brachytherapy, External beam radiotherapy

## Abstract

**Background:**

The management of locally recurrent gynecological carcinoma remains a challenge due to the limited availability of data. This study aims to share our institutional experience in using definitive radiotherapy (RT) for the treatment of locally recurrent cervical and endometrial carcinoma.

**Methods:**

The study retrospectively reviewed 20 patients in our hospital completing salvage 3D image-based HDR brachytherapy, with or without EBRT, for locally recurrent cervical and endometrial carcinoma after surgery. The Kaplan–Meier method was applied to estimate the disease-free survival (DFS) and overall survival (OS). The toxicities were assessed by CTCAEv5.

**Results:**

During a median observation period of 21 months, the study reported a tumor objective response rate of 95%. The 3-year DFS and OS rates were 89.4% and 90.9%, respectively. The EBRT combined with brachytherapy achieved a median cumulative dose of 88 Gy to CTV D90. 14 patients received concurrent and/or systemic chemotherapy. Two patients suffered locoregional recurrence after salvage treatment, one of whom only received salvage brachytherapy for prior RT history. The analysis identified significant predictors for DFS, including tumor histology and FIGO stage. 5 patients observed acute grade 1–2 rectal (15%) or genitourinary (10%) toxicities. Late toxicities including grade 1–2 rectal bleeding (10%) and grade 2 pelvic fracture (5%) were seen in 3 patients.

**Conclusions:**

3D image-guided brachytherapy combined with EBRT shows effective tumor control and acceptable toxicity profile for women with locally recurrent gynecologic cancer. The success in managing vaginal recurrence is notably influenced by histologic subtype and FIGO staging.

## Introduction

Cervical cancer and endometrial cancer are the two main tumor types in gynecological oncology, with a long-term high incidence and mortality [1]. In many low-income and middle-income countries, cervical cancer shows slowly reduced or even increased incidence and mortality, which results in heavy health and economic burden [2, 3]. Meanwhile, endometrial cancer demonstrates a continued rise in incidence worldwide and has the fastest increasing morbidity in women [1, 4]. For patients with early-stage disease, radical surgery is recommended, and subsequent adjuvant treatment is determined according to the pathology. While there are still approximately 5-10% of patients suffered postoperative locoregional recurrence [5–8]. Considering the feasibility of surgical resection, definitive radiotherapy (RT) involving external beam radiotherapy (EBRT) and brachytherapy is more widely used for locally recurrent patients without previous RT history. After treatment for relapse, long-term disease-free survival rates of approximately 40-70% have been reported in some situations [9–11].

Recently, advances in radiotherapy have achieved superior dosimetric outcomes and reduced toxicity. Application of 3D image-guided brachytherapy, especially interstitial brachytherapy, facilitates a higher dose to the clinical target volume (CTV) while minimizing the dose to adjacent organs at risk (OARs) [12, 13]. Herein, we retrospectively reviewed the clinical outcomes and toxicities of twenty patients with postoperative localized recurrence of gynecological tumors after undergoing 3D image-based high-dose-rate (HDR) brachytherapy with (or without) EBRT and chemotherapy. We also evaluated the prognostic factors associated with outcomes after salvage therapy to help understand the disease development and make individualized therapies.

## Methods and materials

### Study design

All patients treated in our hospital from October 2020 to September 2023 for locally recurrent cervical or endometrial cancer after surgery were retrospectively reviewed. The inclusion criteria include: (1) pathologically confirmed vaginal recurrence with no evidence of distant metastasis; (2) finishing HDR-brachytherapy delivered in at least four fractions, with or without external beam radiotherapy. This study was approved by the institutional ethics committee of Shanghai Sixth People’s Hospital.

### Treatment modalities for recurrence

For patients without prior pelvic radiation, definitive RT consisted of EBRT with intensity-modulated radiation therapy (IMRT) and 3D image-based HDR brachytherapy was applied. If the patients have completed postoperative pelvic EBRT before, only brachytherapy was applied. Moreover, concurrent cisplatin (40mg/m^2^, weekly) and/or systemic therapy involving platinum and paclitaxel chemotherapy were also performed based on the physical status of patients during treatment. EBRT was up to a dose of 45–50.4 Gy to the entire vagina plus pelvic lymph nodes at 1.8 Gy per fraction in 25–28 fractions. The CTV for EBRT encompasses the gross tumor, entire vagina, and pelvic lymph nodes including the common iliac, external iliac, internal iliac, obturator, and presacral nodal. Inguinal lymph nodes were also involved for distal vaginal lesions. As to the patients with vaginal plus lymph node recurrence, IMRT delivered a simultaneous integrated boost to 61.6 Gy at 2.2 Gy per fraction to the involved lymph nodes. After EBRT, the patients received image-based HDR brachytherapy at a dose of 24–35 Gy to the vaginal recurrence at 4–6 Gy per fraction in 4–6 fractions. Intracavity brachytherapy by ovoids or cylinder and/or interstitial brachytherapy by free-hand needles was chosen according to patients and tumor anatomy, referring to the extent of disease on pretreatment CT or MRI imaging. All brachytherapy fractions were individually optimized to the CTV with 3-dimensional CT-based planning. The high-risk CTV for brachytherapy involves the residual tumor and entire vaginal surface. Radiation was delivered using an after-loaded 192Ir source in two fractions per week.

### Follow-up and statistical analysis

Outcome measures of disease status included disease-free survival (DFS) and overall survival (OS). DFS was defined as the time from the start of the salvage radiation therapy to the last follow-up attendance or any disease recurrence. OS was defined as the time from the start of salvage radiation therapy to the last follow-up attendance or death from any cause. Besides, we assessed and recorded the treatment-related toxicities by Common Terminology Criteria for Adverse Events version 5 (CTCAEv5). Cumulative DFS and OS were calculated by the Kaplan–Meier method. Univariate survival curve comparisons were performed by the log-rank test to identify prognostic factors for outcomes. All statistical analyses were carried out with GraphPad v9.4.0.

## Results

### Patients and treatment characteristics

A total of twenty patients were reviewed and analyzed. The median age of patients undergoing initial surgery was 55 years (range 32–75). Cervical cancer was the predominant cancer type (*n* = 17). According to the surgical histology, most lesions were precancerous or early-stage cancer (FIGO I). One patient received postoperative pelvic EBRT at a dose of 50.4 Gy in 28 fractions and concurrent nedaplatin at 40 mg weekly for 5 cycles as adjuvant treatment. Besides, three patients accepted adjuvant platinum and paclitaxel chemotherapy alone for 2–3 cycles after surgery. More characteristics of patients and initial therapy are presented in Table 1.

**Fig. 1:**
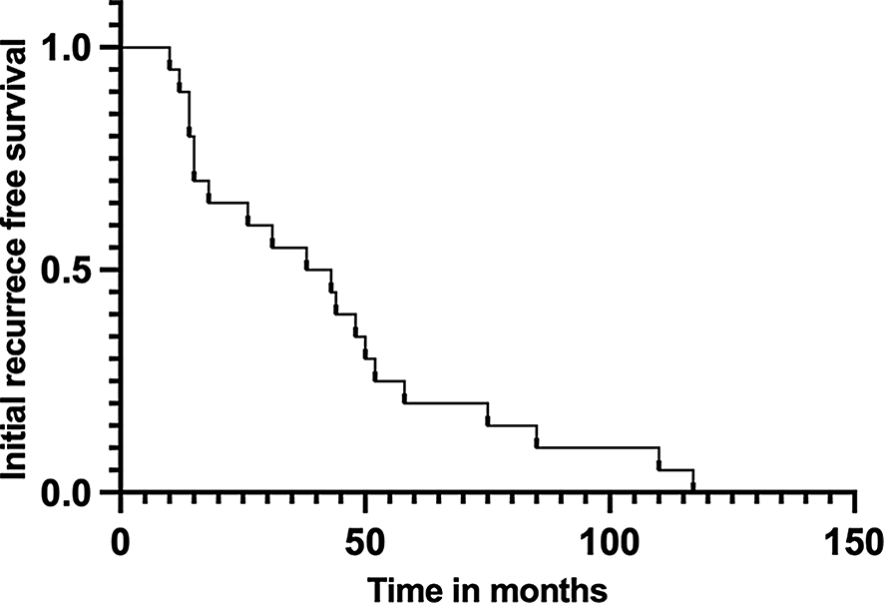
Initial recurrence-free survival of patients in the study

The median time from primary hysterectomy to vaginal recurrence was 40.5 months (range 10.0–117.0) (Fig. 1). 80% of recurrences (*n* = 16) were limited to the vagina, the rest (*n* = 4) involved both vagina and pelvic or inguinal lymph nodes. Except for one patient who only received salvage brachytherapy because of previous RT history, all other patients (*n* = 19) received definitive RT involving pelvic EBRT and 3D image-guided HDR vaginal brachytherapy. Based on patient anatomy and the residual tumor size and extension, 55% of patients (*n* = 11) had intracavity brachytherapy, 35% of patients (*n* = 7) had interstitial brachytherapy, and the rest (*n* = 2) had hybrid brachytherapy including both. The salvage radiation treatment achieved a median dose of 88 Gy to 90% clinical target volume (D90) in the equivalent dose in 2 Gy fractions (EQD2). The cumulative dose of CTV D90 was above 75 Gy in all patients except one only receiving brachytherapy. Along with radiotherapy, concurrent cisplatin and/or systemic therapy was performed in 70% of patients (*n* = 14). The other details of the salvage treatment are listed in Table 2.

### Clinical outcomes and prognostic factors

**Fig. 2:**
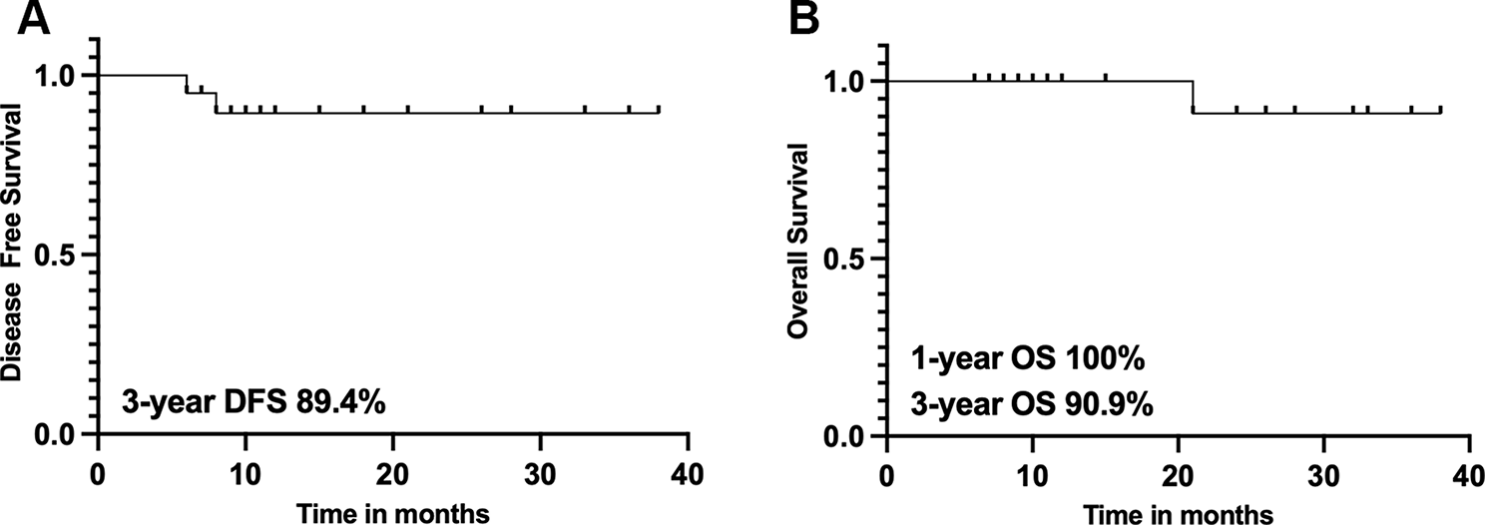
Kaplan-Meier estimates of (**A**) DFS and (**B**) OS for patients with vaginal recurrence after salvage therapy

After salvage treatment, the objective response rate was 95%. With a median follow-up time of 21 months (range: 3–38), the 3-year DFS and OS were 89.4% and 90.9%, respectively (Fig. 2A-B). Two patients (10%) had locoregional recurrence at 6 and 8 months after salvage treatment, and one of them only received salvage brachytherapy because of prior pelvic RT history. No distant failure was found during the follow-up.

**Fig. 3:**
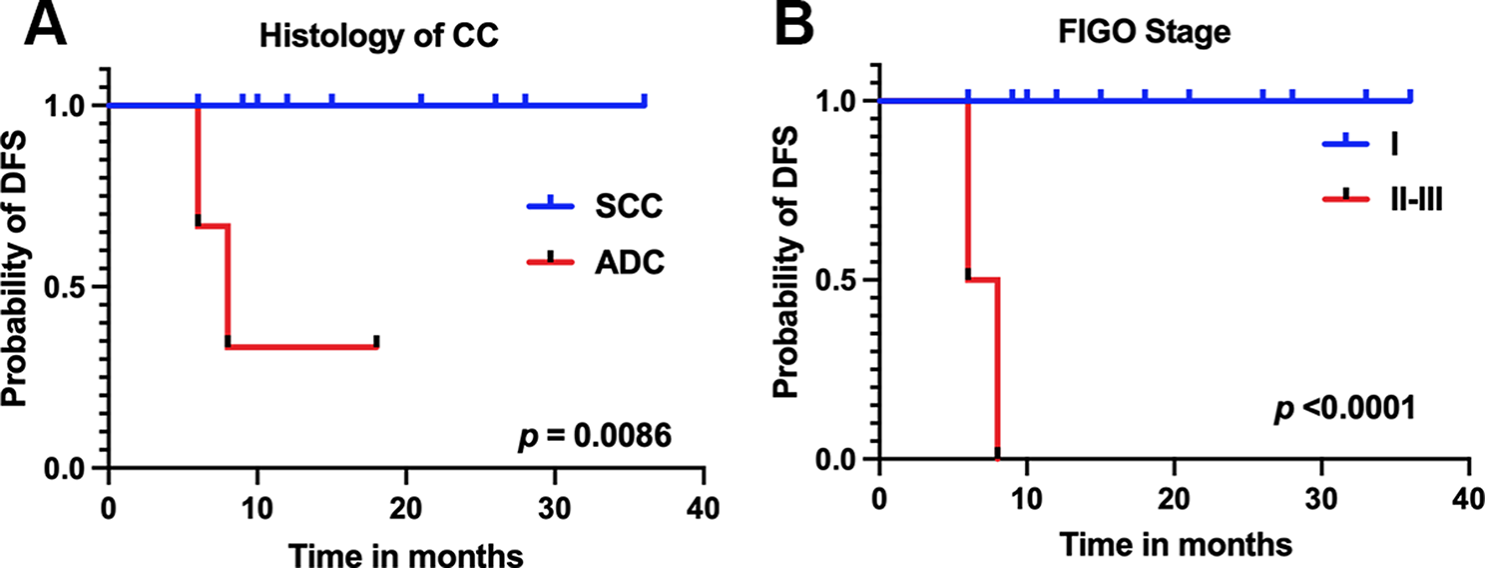
The DFS curves stratified by (**A**) histologic subtype and (**B**) FIGO stage at initial diagnosis

On univariate analysis for disease control, histology of cervical cancer, FIGO stage after initial surgery were significantly prognostic for DFS (Table 3, all *p* ≤ 0.005). Patients with cervical adenocarcinoma (ADC), higher FIGO stage showed worse outcomes after salvage therapy (Fig. 3A-B). Meanwhile, age at recurrence, the interval between initial surgery and recurrence, tumor grade, pelvic nodal recurrence, brachytherapy type, and chemotherapy use had no predictive value for DFS (Table 3, all *p* > 0.1).

### Treatment-related toxicity

There was no acute grade 3 or higher toxicity observed. 15% of patients (*n* = 3) had acute grade 1–2 rectal toxicities characterized by diarrhea or rectal hemorrhage. Other 10% of patients (*n* = 2) had acute grade 1–2 cystitis or urinary tract pain. Late toxicities, including grade 1–2 rectal hemorrhage (10%) and grade 2 pelvic fracture (5%, hairline fracture), were observed in three patients.

**Table 1:** Initial clinical and treatment characteristics

Initial clinical characteristics	Patients (*n* = 20)
Median age (years)	55 (32–75)
Lesion sites and histology	
Uterine cervix	17 (85%)
CIN3	5 (25%)
Squamous cell carcinoma	9 (45%)
Adenocarcinoma	3 (15%)
Uterine corpus	3 (15%)
Endometrioid adenocarcinoma	2 (10%)
Adenocarcinoma with squamous differentiation	1 (5%)
FIGO stage	
I	13 (65%)
II	1 (5%)
III	1 (5%)
IV	0
Tumor grade	
1	3 (15%)
2	8 (40%)
3	2 (10%)
Unknown	2 (10%)
Initial surgery	
Hysterectomy	1 (5%)
Hysterectomy and adnexectomy	5 (25%)
Hysterectomy, adnexectomy and pelvic lymphadenectomy	14 (70%)
Lymphovascular space invasion	
Negative	5 (25%)
Positive	2 (10%)
Unknown	13 (65%)
Initial strategy after surgery	
Observation	16 (80%)
EBRT with concurrent chemotherapy	1 (5%)
Adjuvant chemotherapy	3 (15%)

*Abbreviation* CC: Cervical cancer; SCC: Squamous cell carcinoma; ADC: Adenocarcinoma; DFS: Disease free survival

**Table 2:** Recurrence characteristics and salvage therapy parameters

Recurrence characteristics and salvage therapy parameters	Patients (*n* = 20)
Median age at recurrence (years)	58 (34–78)
Median time to recurrence (months)	40.5 (10–107)
Type of recurrence	
Insolated vaginal	16 (80%)
Vaginal and lymph node	4 (20%)
Salvage radiotherapy	
EBRT and brachytherapy	19 (95%)
Brachytherapy	1 (5%)
Brachytherapy type	
Intracavity	11 (55%)
Interstitial	7 (35%)
Intracavity and Interstitial	2 (10%)
Pelvic EBRT prescription	
45 Gy in 25 fractions	6 (30%)
50.4 Gy in 28 fractions	13 (65%)
Median HDR brachytherapy prescription (Gy)	30 (24–35)
Median number of brachytherapy fractions	5 (4–6)
Median total dose to CTV (D90) in EQD2 (Gy)	88 (32–102)
Median D2cc for OARs (Gy)	
Bladder	64.6 (12.6–74.3)
Rectum	62.4 (10.7–71.1)
Bowel	52.2 (1.9–66.3)
Concurrent and/or systemic therapy at salvage	14 (70%)
Concurrent cisplatin	4 (20%)
Systemic therapy	4 (20%)
Concurrent and systemic therapy	6 (30%)

*Abbreviation* EBRT: external beam radiotherapy; HDR: high dose rate; CTV: clinical tumor volume; EQD2: equivalent dose in 2Gy fractions; Gy: gray; D2cc: dose delivered to 2-cc volume; OARs: organs at risk

**Table 3:** Prognostic factors for PFS following salvage therapy for the vaginal recurrence

Factors	Patients	*p* value
Age at recurrence		0.30
≤60 years	13	
>60 years	7	
Interval between initial surgery and recurrence		0.104
≤36 months	9	
>36 months	11	
Histology of cervical cancer		0.009*
Squamous cell carcinoma	9	
Adenocarcinoma	3	
FIGO stage		*p* < 0.0001*
I	13	
II-III	2	
Tumor grade		0.41
1	3	
2–3	10	
Pelvic nodal recurrence		0.74
Negative	16	
Positive	4	
Brachytherapy type		0.24
Intracavity	11	
Interstitial	7	
Intracavity and Interstitial	2	
Chemotherapy use		0.46
No	6	
Yes	14	

*Abbreviation* EBRT: external beam radiotherapy; CIN3: Cervical intraepithelial neoplasia 3; FIGO: International Federation of Obstetrics and Gynecology

## Discussion

In this cohort of 20 patients with vaginal recurrence of cervical or endometrial cancer, salvage image-guided HDR brachytherapy with or without EBRT resulted in excellent tumor control and overall survival. The local failure rate was 10%. The 3-year DFS and OS were 89.4% and 90.9%, respectively. Meanwhile, the patients did not experience severe toxicities after salvage treatment. Only grade 1–2 acute or late gastrointestinal, genitourinary toxicities and pelvic insufficiency fracture were observed during the follow-up.

The patients in our cohort showed better survival outcomes than those reported in other studies [10, 14–20]. For patients with local recurrence who received salvage brachytherapy with or without EBRT, the 3-year DFS and OS were approximately 34-55% and 57-75%, respectively [17, 18, 20]. While for those who completed both EBRT and brachytherapy, the 3-year DFS and OS increased to 68–75% and 67–80%, respectively [10, 15, 19]. In our study, most patients (95%) received EBRT and sequential brachytherapy. More importantly, the median cumulative dose to CTV D90 (88 Gy, EQD2) was higher than that in previous reports (70–82 Gy, EQD2) [10, 11, 14–20], which accounted for the better outcomes. During the follow-up, none of the patients suffered distant metastasis, which might be attributed to the use of concurrent and /or systemic chemotherapy.

Analysis of prognostic factors associated with DFS demonstrated cervical ADC, relatively advanced tumor stage (FIGO II-III) predicted worse outcomes after salvage treatment. It has been reported that patients with ADC had a poorer prognosis than squamous cell carcinoma in the uterine cervix [21, 22]. Our finding reconfirmed that ADC was a significant prognostic factor affecting relapse-free survival of patients with locally recurrent cervical cancer. The prognostic role of the FIGO stage was consistent with prior studies [10, 15].

In the present study, with a median dose delivery of 88 Gy prescribed to CTV, no patients suffered grade 3 or higher toxicities. The median D2cc for the bladder, rectum, and bowel were 64.6 Gy, 62.4 Gy and 52.2 Gy, respectively. According to the dose constraint recommended by ASTRO clinical practice guideline [23], ideal dose constraints for the bladder, rectum, and bowel were achieved in 100%, 60%, and 95% of the patients in the study. No one had the dose of OARs exceeded the maximum dose constraints. Limitations of this study included its retrospective nature and small sample size, which resulted in the preclusion of multivariate analysis. Nevertheless, our data contributed to the limited number of related series in the literature, as well as shared our institutional experience in exploring treatment modalities and dose prescriptions for treating isolated vaginal recurrence.

## Conclusion

In conclusion, our study indicates that image-guided brachytherapy combined with EBRT could achieve favorable tumor control and tolerable treatment-related toxicities in patients with locoregionally recurrent gynecological cancer. Tumor histology and FIGO stage revealed prognostic value for clinical outcomes.

## Data Availability

The datasets used and/or analysed during the current study are available from the corresponding author on reasonable request.
